# Avascular osteonecrosis in kidney transplant recipients: Risk factors in a recent cohort study and evaluation of the role of secondary hyperparathyroidism

**DOI:** 10.1371/journal.pone.0212931

**Published:** 2019-02-22

**Authors:** Renaud Felten, Peggy Perrin, Sophie Caillard, Bruno Moulin, Rose-Marie Javier

**Affiliations:** 1 Department of Rheumatology, University Hospital, Strasbourg, France; 2 Fédération de Médecine Translationnelle (FMTS), Strasbourg, France; 3 Department pf Nephrology-Transplantation, University Hospital Strasbourg, France; Weill Cornell Medicine, UNITED STATES

## Abstract

Avascular osteonecrosis (AVN) is a bone complication that indicates poor functional prognosis. Modern immunosuppressive and steroid-sparing drugs have significantly lowered the occurrence of AVN after kidney transplantation (KT). However, recent data on its incidence rates and risk factors are lacking. Using a large, recent cohort, we sought to investigate AVN incidence and risk factors, with a special focus on mineral and bone disorders. We conducted a cohort study in 805 patients who underwent KT between 2004 and 2014. AVN was identified in 32 patients (4%): before KT in 15 (1.8%) and after KT in 18 (2.2%) cases, including one patient with both. In the group with post-KT AVN, the median time intervals from KT to 1) first symptoms and 2) AVN diagnosis were 12 months [1–99] and 20 months [4–100], respectively. Being overweight/obese, having pre-transplant diabetes or hyperparathyroidism at transplantation, developing acute rejection, and receiving higher cumulative corticosteroid doses were associated with AVN occurrence. Multivariate analysis revealed that BMI ≥ 26 kg/m^2^ and higher cumulative corticosteroid doses were predictive of AVN. In conclusion, overweight/obesity is a strong risk factor for AVN. Despite a low maintenance dose, the use of corticosteroids—mostly for treatment of acute rejection—remains an independent risk factor.

## Introduction

Avascular osteonecrosis (AVN) is a disabling bone complication that can occur after kidney transplantation (KT). Before the use of modern immunosuppressive drugs, AVN developed in approximately one third of post-transplant patients [1,2]. Corticosteroids play a central role in the genesis of AVN, and recent reports suggest that the use of steroid-sparing anti-calcineurin agents has reduced incidence rates to less than 5% [3–5]. There is a lack of recent data regarding incidence rates, clinical features, or AVN risk factors, as most studies evaluate few risk factors in a limited population. Few studies concerning recipients with renal transplant performed after the year 2000 are available [6–10].

The underlying pathophysiological mechanism that leads to AVN is a diminished blood flow to the bone, leading to necrosis and bone destruction. The femoral head is the most commonly affected region, followed by other weight-bearing long bones. Optimal treatment aims to prevent the collapse of affected bones. This makes identifying high-risk patients and diagnosing AVN in the earlier stages essential. Some risk factors are already known to associate with AVN in both the general population [11] and kidney transplant (KT) recipients, such as corticosteroids [7,12–14], alcohol consumption [15], dyslipidemia [16], and hemostatic disorders [6,17]. Several other risk factors are also suspected in the KT population, such as osteopenia [8,12,15] and secondary hyperparathyroidism [2,6].

To address this, we conducted a retrospective study using a large and recent cohort of KT patients to determine AVN incidence rates, disease-related characteristics, and patient-associating factors. We particularly focused on the role of secondary hyperparathyroidism and pre-transplant bone mineral density (BMD) in AVN occurrence.

## Patients and methods

### Study population

In this retrospective cohort study, we included all adult patients who had undergone KT between January 2004 and June 2014 in the Nephrology-Transplantation Department of Strasbourg University Hospital, Strasbourg, France (n = 888). Subjects with multi-organ transplantation (n = 44), or incomplete data during the first three months (n = 39), were excluded. Patients were followed after KT until June 2016. This period was selected to allow a minimum follow-up period of two years after transplantation.

The Institutional Review Board of Strasbourg University Hospital approved the collection of cohort data and subsequent analysis in the study (approval number: DC-2013-1990) and waived the requirement for informed consent. All data were fully anonymized before we accessed them. The transplantation database of our center was used to collect patient characteristics and potential risk factors for AVN. AVN was established by cross-referencing the transplantation database, the university hospital diagnostic database, the radiological data set, and medical records. AVN was only collected if a diagnosis was confirmed by imaging. For each case of AVN, the associated patient medical record was reviewed and AVN characteristics recorded, including date of symptom onset, presence before KT, the type and number of affected sites, and surgical treatment performed. During the follow-up period, delayed graft function (use of dialysis or creatininemia > 250 μmol/L at day 7 after KT), occurrence of biopsy-proven acute rejection, and immunosuppressive treatments were recorded.

With regards to the immunosuppressive treatments, immunologically high-risk recipients were treated with thymoglobulin, tacrolimus, mycophenolate mofetil (MMF), and steroids, and immunologically low-risk patients received basiliximab, cyclosporine (CsA), MMF, and steroids. At induction, corticosteroid boluses (2×250 mg) were administered. Prednisone was given at 1 mg/kg/day (with a maximal limit dose of 80 mg/day) during the first week and then gradually tapered and discontinued between the third and sixth month, except in patients with specific immunological risks or acute rejection (who were maintained at 0.1 mg/kg/day). Target trough levels of tacrolimus were 8 to 12 ng/mL in the first 6 months, and 6 to 8 ng/mL thereafter. Target trough levels of CsA were 150 to 200 ng/mL in the first 6 months, 125 to 150 ng/mL from 6 to 12 months, and 75 to 125 ng/mL thereafter. Initial dose of MMF was 3 g by day in association with CsA and 2 g by day in association with tacrolimus, which was adapted for a target of M3 MMF AUC between 30 to 60 h.mg/L. In cases of acute cellular rejection, steroid pulses (500 mg) were administered for 3 days, followed by oral steroids at a dose of 1 mg/kg per day, and a switch to tacrolimus in patients treated with CsA. CNI trough levels were not adjusted during rejection episodes. The management of our patients was not changed throughout our study. The cumulative doses of oral and bolus corticosteroids were calculated during the first three months and the first year.

### Bone and mineral metabolism evaluation

Various biochemical parameters were prospectively determined at transplantation (before the surgical transplant procedure) and in the routine post-transplant follow-up. Serum PTH levels were measured using intact PTH assays (Elecsys; Roche, reference range: 15–65 pg/mL replaced by Centaur, Siemens; reference range 11–79 pg/mL since June 2013). Estimated glomerular filtration rates (eGFRs) were calculated using the four-variable equation derived from the Modification of Diet in Renal Disease (MDRD) Study. Radioimmunoassays were used to determine the serum 25OH-vitamin D (Diasorin, Stillwater), bone alkaline phosphatase serum (Access Ostase; Beckman; reference range: 2.2–14.5 μg/L), and C-telopeptide serum levels (Elecsys; Roche; reference: < 0.54 μg/L). Pre-transplant bone mineral density (BMD) was measured during routine pre-transplant evaluation using a dual-energy X-ray absorptiometry placed at the lumbar spine, femoral neck, and total hip. Following the World Health Organization criteria [18], osteopenia was defined as a T-score between -1 and −2.5, whereas osteoporosis was defined as a T-score < −2.5.

### Statistical analysis

Data are shown as the means ± standard deviation, the medians [minimum–maximum] or (IQR1-IQR3), or the percentages for parametric and nonparametric parameters, and categorical variables, respectively. Normality was tested using Shapiro-Wilk tests. In order to limit biases, patients with AVN before transplantation (PreT-AVN group) and patients with new onset of AVN after transplantation (NOAVN group) were analyzed separately and compared to patients without AVN. Variables between groups were compared using Student’s *t*-tests, Mann-Whitney U-tests, Wilcoxon test, Fisher tests, or chi-square tests, as appropriate. Association factors and the occurrence of post-transplant development of NOAVN were tested using log-rank tests. The time of AVN onset was assumed to be the date of AVN diagnosis, with patients censored at death, loss to follow up, kidney loss, or at the end of the follow-up period. Correlations were assessed by linear regression, Spearman’s test, or Pearson’s test as appropriate.

Potential predictive risk factors that showed an association with a p < 0.2 in the univariate analysis were examined as covariates in a multivariable Cox regression model (covariates: gender, pre-transplant diabetes, BMI ≥ 26 kg/m^2^ at KT, PTH > 300 pg/mL at KT, cumulative corticosteroid dose at M3, and acute rejection if occurred before AVN event). The 26 kg/m^2^ threshold for the BMI was chosen based on BMI analysis using a receiver operating characteristic (ROC) curve. The 300 pg/mL threshold for PTH at time of transplantation was chosen as this had been independently shown to associate with fractures after KT [19]. This threshold also associates with histomorphometric signs of high-turnover bone disease in dialysis patients [20]. Values of p < 0.05 were considered statistically significant. The analyses were performed using SAS JMP Software 7.1 (SAS Institute Inc).

## Results

### Patient characteristics and immunosuppressive treatments

In total, 805 patients were analyzed at both transplantation and month 3 (M3), 784 at M12, 742 at M24, and 433 at M60. Among 805 KT, 184 cases were re-transplants (22.9%) as compared to 621 primary KT. The median post-transplant follow-up was 67 months [range 3–150], and 89.6% were deceased donor transplantations. An overview of patient characteristics and outcomes after transplantation is shown in Table 1. Specifically, the cohort was 61% males and 39% females, 95% Caucasians, the median age was 51 years, and 25% of KT recipients had pre-transplant diabetes.

**Table 1:** Summary of patient characteristics at the time of kidney transplantation (n = 805) and details concerning immunosuppressive treatments and acute rejection.

Male (%)/Female (%)	492 (61.1)/313 (38.9)
Age at transplantation (years)	51.0 [18.0–83.0]
Primary kidney disease (%)	
• Glomerulonephritis	33.9
• Diabetic nephropathy	9.3
• Interstitial nephropathy	36.4
• Vascular nephropathy	7.6
• Other/unknown	12.8
Dialysis duration (months)	33 [0–452]
HD/PD/preemptive transplantation (%)	70.6/19.2/10.2
Transplantation rank ≥ 2 (%)	22.9
Pre-transplant corticotherapy, n = 419 (%)	59.6
Body mass index (kg/m^2^)	24.7 [13.6–39.1]
Pre-transplant diabetes Type 1/Type 2	1.4/23.9
Dyslipidemia, n = 758 (%)	50.4
Current or former smoker, n = 274 (%)	59.9
Pre-transplant BMD status, n = 707 (%):	
• Normal/osteopenia/osteoporosis	31.3/45.8/22.9
Immunosuppressive treatments	
• Anti-IL2 receptor antibody/Thymoglobulin (%)	36.7/58.3
• Cyclosporin A/Tacrolimus/mTOR inhibitor (%)	60.7/38.5/1.2
• Total (oral + bolus) corticosteroid dose at M3 (mg)	2075 [500–4790]
• Total (oral + bolus) corticosteroid dose at M12 (mg)	2978 [500–9855]
Delayed graft function (%)	28.5
Acute rejection	
• Cumulative incidence at M3/M12/M24 (%)	7.7/16.2/19.8
• Pure TCMR/pure ABMR/mixed (%)	14.9/2.2/2.5

Data are given as percentages or medians [minimum–maximum], as appropriate. HD, hemodialysis; PD, peritoneal dialysis; BMD: bone mineral density; mTOR, mammalian target of rapamycin; n, number of analyzed patients; TCMR, T cell-mediated rejection; ABMR, antibody-mediated rejection.

### Incidence rates and AVN characteristics

**Fig 1:**
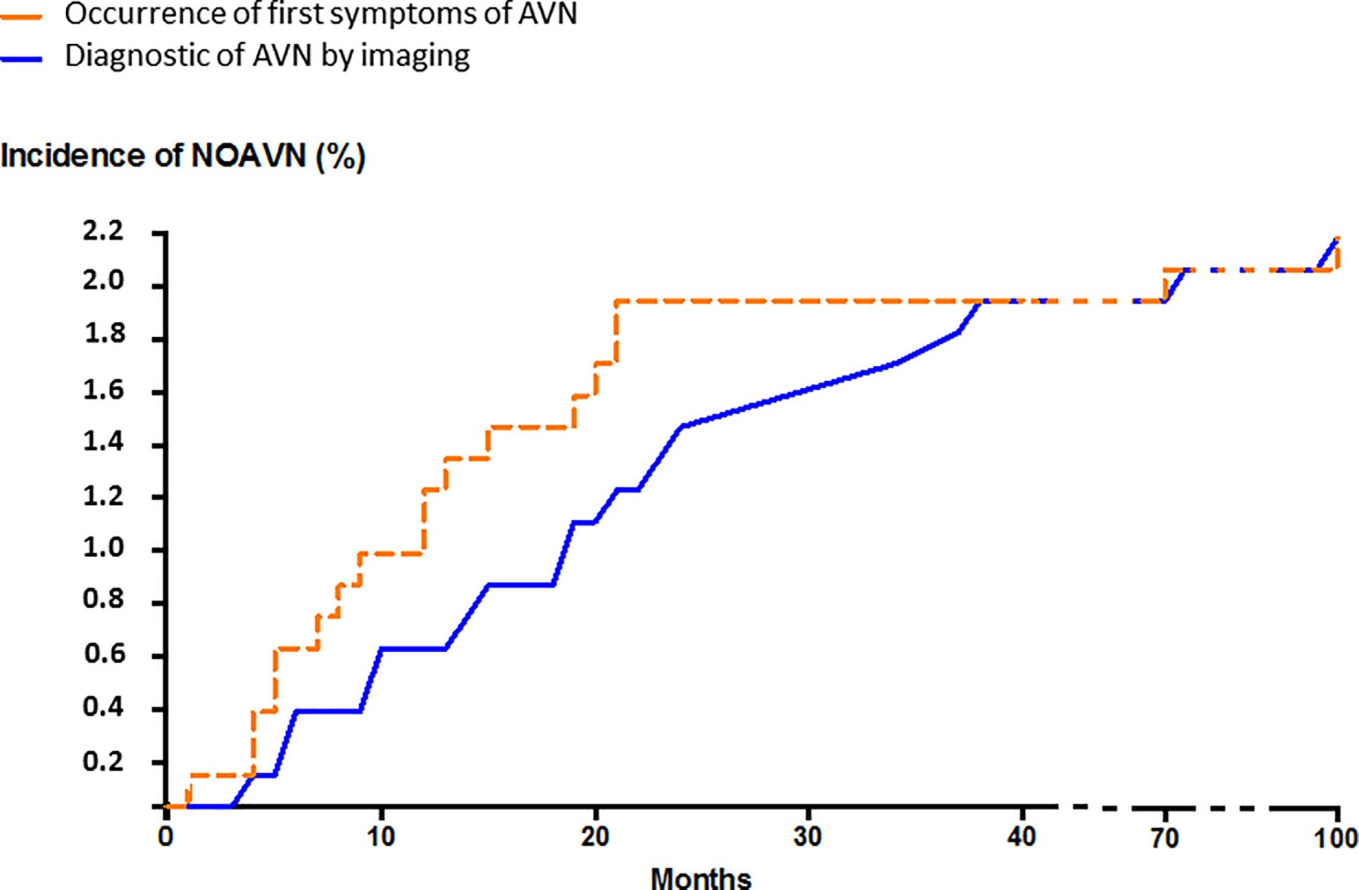
A Kaplan-Meier plot of the time to first symptoms of new onset of avascular osteonecrosis (NOAVN) and to NOAVN diagnosis after kidney transplantation.

We identified AVN in 32 recipients, leading to an overall prevalence rate of 3.98%. AVN had developed before transplantation in 15 patients (1.86%) and after transplantation in 18 patients (2.24%). One patient displayed both pre- and post-transplantation AVN. The rate of NOAVN among the primary transplant cases was 2.4% (15/621). The post-transplant incidence was 0.6% at M12 and 1.5% at M24 (Fig 1).

Twelve of 15 pre-transplant AVN cases (80%) had received a previous transplant. (Table 2). PreT-AVN patients were more likely to have been treated with corticosteroids before transplantation (p = 0.006), more likely to have had a longer duration of dialysis (p < 0.001), and more often received a second or further transplant (p < 0.0001) compared to patients without AVN (Table 2).

**Table 2:** Summary of patient characteristics at the time of kidney transplantation, according to AVN diagnosis.

	Patients without AVN n = 773	New Onset AVN group n = 18	p^a^	Pre-transplant AVN group n = 15	p^b^
Male (%)/Female (%)	468 (60.5)/305 (39.5)	14 (77.8)/4 (22.2)	0.151	11 (73.3)/4 (26.7)	0.42
Age at transplantation, years [range]	51.0 [18–83]	54.5 [24–72]	0.69	52.0 [33–66]	0.26
Primary kidney disease (%)			0.64		0.33
• Glomerulonephritis	35.7	44.4		60	
• Vascular nephropathy	7.5	11.1		6.7	
• Diabetic nephropathy	9.7	0		0	
• Interstitial nephropathy	34.0	33.3		26.7	
• Other or unknown	13.1	11.2		6.7	
Dialysis duration, months [range]	33 [0–422]	39 [16–161]	0.36	189 [17–452]	< 0.001
Preemptive KT/PD/HD (%)	10.2/19.7/70.1	0/11.1/88.9	0.185	20.0/6.7/73.3	0.26
Deceased donor (%)	89.1	100	0.24	100	0.39
Transplantation rank ≥ 2 (%)	22.9	16.7	0.77	80	< 0.001
Pre-transplant corticotherapy, n = 419 (%)	227 (58.7)	10 (55.6)	0.81	14 (93.3)	0.006
BMI (kg/m^2^) (IQR1–IQR3)	24.7 (21.6–28.1)	27.6 (26.0–32.6)	0.003	22.2 (19.3–25.3)	0.140
BMI ≥ 25 kg/m^2^ (%)	360 (46.6)	17 (94.4)	< 0.001	6 (42.9)	0.9
BMI ≥ 30 kg/m^2^ (%)	108 (14.0)	6 (33.3)	0.020	0	0.245
Pre-transplant Diabetes (%)	191 (24.7)	8 (44.4)	0.093	5 (33.3)	0.545
Dyslipidemia, n = 758 (%)	370 (50.9)	8 (44.4)	0.63	4 (26.7)	0.072
Smoker, n = 274 (%)	147 (60.7)	9 (50)	0.45	9 (60)	0.9
HIV seropositivity	3	0	0.9	0	0.9
Pregnancies 0 - ≥ 1, n = 247	38–201	1–3	0.50	0–4	0.9
Sickle cell disease, n = 382 (%)	7 (2)	0	0.9	0	0.9
Hemostasis disorders, n = 669 (%)	171 (26.8)	7 (41.1)	0.28	2 (13.3)	0.37
Serum level of PTH, median (IQR1–IQR3), n = 773	202.5 (106.0–382.9)	337.0 (215.5–472.3)	0.042	199.4 (59.2–388.2)	0.51
Serum level of 25-hydroxy-vitamin D median (IQR1–IQR3), n = 292	18.7 (10.7–31.1)	25.1 (15.3–29.8)	0.33	18.0 (5.9–39.6)	0.80
Pre-transplant osteopenia-osteoporosis, n = 707 (%)	313(46.2)–154(22.8)	5(31)–5(31)	0.48	6(40)–3(20)	0.75

p^a^: New Onset AVN group compared to patients without AVN, p^b^: pre-transplant AVN group compared to patients without AVN.Data are given as percentages or medians [minimum–maximum], or (interquartile IQR1-IQR3), as appropriate. AVN, avascular osteonecrosis; BMI, body mass index; HD, hemodialysis; HIV, human immunodeficiency virus; KT, kidney transplantation; n, number of analyzed patients; PD, peritoneal dialysis

Among the patients with new onset AVN after transplantation (NOAVN group, n = 18), 14 (78%) were males. The median age at diagnosis was 50 years [range 25–75]. The median times between transplantation to the appearance of articular pain and AVN diagnosis were 12 months [range 1.2–99.2 months] and 20 months [3.9–99.8], respectively. The median delay between first symptoms and diagnosis was 3.4 months [0.3–34.5] (Fig 1). In 88% of cases, the first symptoms appeared within 24 months of transplantation. MRI and computed tomography confirmed diagnosis in 13 and three patients, respectively. Radiographs and bone scintigraphy confirmed AVN in one patient each. In a single patient, AVN was asymptomatic and was diagnosed using a computed tomography scan performed for another reason. At diagnosis, 10 (55.6%) had localized regions of AVN at a minimum of two different sites. These AVN sites included the femoral head (15 patients), the femoral condyle (two patients), and the ankle (one patient). Seven patients received a pamidronate infusion to improve pain and function. Nine patients were treated with total hip arthroplasty during the follow-up period.

### Comparison of patients with NOAVN and without AVN

**Fig 2:**
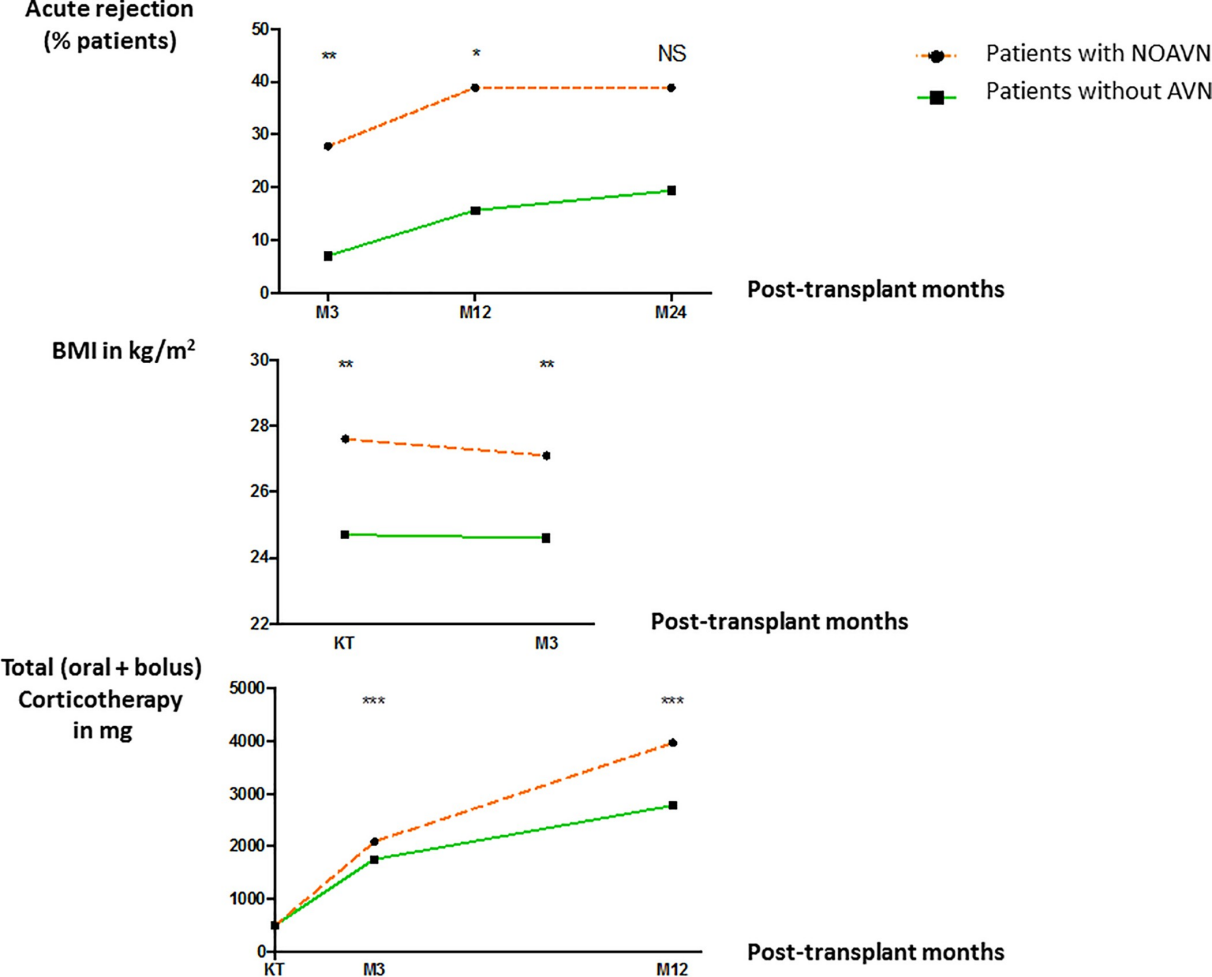
Occurrence of new onset of avascular osteonecrosis (NOAVN), according to acute rejection, BMI, and cumulative corticosteroid dose. NS, not significant; *, p<0.05; **, p<0.01, ***, p<0.001.

Various factors were compared between patients with NOAVN and those without any AVN (Tables 2 and 3). Patients with NOAVN were characterized by a higher BMI, higher acute rejection rate, and greater cumulative doses of corticosteroids (Fig 2). Oral cumulative doses at M3 were higher in the NOAVN group (Table 3). Serum levels of PTH and phosphorus at transplantation were also higher in the NOAVN group than in patients without AVN (Table 4). Pre-transplant BMD statuses were comparable between groups.

**Table 3:** Outcomes and immunosuppressive treatments after kidney transplantation, according to new onset AVN diagnosis (patients with pre-transplant AVN were excluded).

	Patients without AVN n = 773	NOAVN group n = 18	p
Follow-up duration, months [range]	64.7 [3–149.9]	55.2 [28.1–143.9]	0.85
Delayed graft function (%)	217 (28.1)	8 (44.4)	0.128
Acute rejection (TCMR and/or ABMR)			
• At M3 (%)	55 (7.1)	5 (27.8)	0.008
• At M12 (%)	118 (15.7)	7 (38.9)	0.016
• At M24 (%)	138 (19.4)	7 (38.9)	0.065
Body mass index at M3 (kg/m^2^) (IQR1–IQR3)	24.6 (21.8–27.9)	26.9 (26.2–30.9)	0.003
Diabetes after transplantation (%)	311/702 (44.3)	10 (55.6)	0.35
Dyslipidemia after transplantation (%)	460/667 (69.0)	12 (66.7)	0.801
Immunosuppressive regimen at induction			
• Anti-IL2 receptor antibody (%)	282 (36.6)	10 (55.6)	0.099
• Thymoglobulin (%)	451 (58.5)	8 (44.4)	0.23
• Cyclosporin (%)-Tacrolimus (%)	470 (61.3)-297 (38.7)	14 (77.8)-4 (22.2)	0.15
Corticosteroid dose (IQR1–IQR3)			
• Oral cumulative dose at M3, mg	1758 (1348–2078)	2094 (2028–2105)	<0.001
• Bolus dose at M3, mg	500 (500–500)	500 (500–500)	0.73
• Total (oral + Bolus) dose at M3, mg	2035 (1675–2578)	2594 (2527–2605)	<0.001
• Oral cumulative dose at M12, mg	2280 (1680–3165)	3453 (3267–4314)	<0.001
• Bolus dose at M12, mg	500 (500–500)	500 (500–2000)	0.008
• Total (oral + Bolus) dose at M12, mg	2780 (2270–3928)	3970 (3767–6314)	<0.001
• Total (oral + Bolus) dose at M12, mg/kg	39.8 (32.3–56.4)	49.7 (44.5–67.9)	0.034
• Use of bolus after discharge in the first year (%)	116 (17.7)	7 (38.9)	0.011

Data are given as percentages or medians [minimum–maximum] or (interquartile IQR1–IQR3), as appropriate. AVN: avascular osteonecrosis; NOAVN: new Onset AVN; M3, M12, M24: 3, 12 and 24 post-transplant months; TCMR: T cell-mediated rejection; ABMR: antibody-mediated rejection

**Table 4:** Biochemical parameters at transplantation and at M3, according to AVN presence.

	Patients without AVN n = 773	New onset AVN group n = 18	p
**Serum level of PTH**			
At transplantation (pg/mL)	202 [0–2095]	337 [78–640]	0.042
PTH < 130 pg/mL (%)	30.9	11.8	0.090
PTH 130–300 pg/mL (%)	35.4	29.4	0.61
PTH > 300 pg/mL (%)	33.7	58.8	0.031
At M3 (pg/mL)	108 [1–1384]	149 [37–418]	0.27
PTH > 130 pg/mL (%)	40.8	55.6	0.20
**Serum level of calcium**			
At transplantation (mg/dL)	9.0 [4.2–12.3]	8.8 [7.4–10.5]	0.50
At M3 (mg/dL)	9.6 [7.3–12.4]	9.5 [8.1–10.8]	0.9
**Serum level of phosphorus**			
At transplantation (mg/dL)	3.1 [1.0–10.1]	3.9 [2.2–6.3]	0.019
At M3 (mg/dL)	2.9 [1.6–7.7]	2.9 [1.7–4.8]	0.66
**Serum level of 25-hydroxy-vitamin D**			
At transplantation (μg/L)	18.7 [2.0–106.0]	25.1 [6.0–57.9]	0.32
At M3 (μg/L)	25.5 [1.4–84.8]	20.6 [11.1–51.6]	0.62
**Serum level of bone alkaline phosphatase**			
At transplantation (μg/L)	14.4 [4.9–190.8]	17.8 [9.0–33.7]	0.57
At M3 (μg/L)	14.4 [4.5–104.4]	12.9 [6.8–28.2]	0.59
**Serum level of C-telopeptide** (μg/L) at M3	0.9 [0.1–3.2]	0.8 [0.2–2.3]	0.65
**eGFR** (mL/min/1.73 m^2^) at M3	48.9 [6.0–128.4]	48.7 [8.7–100.2]	0.69

Data are given as percentages or medians [minimum–maximum], as appropriate. AVN: avascular osteonecrosis, eGFR: estimated glomerular filtration rate (MDRD), M3: post-transplant month three, PTH: intact parathyroid hormone

### Predictive factors of NOAVN

Univariate survival analysis revealed that factors that associated with NOAVN at transplantation were a BMI ≥ 26 kg/m^2^ (p = 0.0004), pre-transplant diabetes (p = 0.040), and PTH > 300 pg/mL (p = 0.044). There was a nonsignificant trend for more AVN in male patients (p = 0.11). AVN was not statistically associated with dyslipidemia (p = 0.57). Factors that associated with NOAVN at M3 were BMI ≥ 26 kg/m^2^ (p = 0.0003), acute rejection in the first three months (p = 0.0045), and a higher cumulative corticosteroid dose in the first three months (p < 0.0001). The multivariate analysis identified higher cumulative corticosteroid dose at M3 and BMI ≥ 26 kg/m^2^ as independent risk factors for NOAVN development (Table 5). There was no statistical difference in new onset AVN after transplantation in re-transplant patients compared to primary transplant recipients.

**Table 5:** Cox multivariate analyses: Factors associated with new onset AVN.

	Univariate HR (CI 95%)	p	Multivariate AHR (CI 95%)	p
Gender (male)	2.32 (0.76–7.04)	0.14	2.07 (0.57–7.48)	0.24
Pre-transplant diabetes	2.76 (1.08–7.01)	0.033	2.27 (0.85–6.08)	0.10
BMI at transplantation ≥ 26 kg/m^2^	4.77 (1.70–13.4)	0.0030	3.30 (1.00–10.86)	0.049
PTH at transplantation > 300 ng/L	2.68 (1.02–7.05)	0.046	2.10 (0.79–5.60)	0.13
Cumulative corticosteroid dose at M3	2.11 (1.52–2.93) *	< 0.0001	1.55 (1.03–2.32) *	0.034
Acute rejection at M24 and before AVN	2.48 (0.93–6.64)	0.070	1.69 (0.55–5.17)	0.35

Model included factors associated with NOAVN in univariate analysis with p < 0.2

AHR: adjusted hazard ratio; AVN: avascular osteonecrosis; BMI: body mass index: M3 and M24: 3 and 24 post-transplant months

* by 500 mg of corticosteroid

Cumulative corticosteroid dose at M12 was positively associated with acute rejection at M12 (p < 0.0001). Cumulative oral and bolus were positively correlated (rho = 0.42, p < 0.0001). BMI ≥ 25 kg/m^2^ at transplantation was associated with PTH > 300 pg/mL at transplantation (p = 0.0002) and with pre-transplant diabetes (p < 0.0001).

## Discussion

Here, we report a recent retrospective cohort study that assessed AVN in a large population of 805 kidney allograft recipients. Our rate of 4% is comparable with most recent studies of symptomatic AVN, with rates ranging between 4 and 7% [4,21–23]. The two-year post-transplant incidence is about 1% and is comparable to the most recent study [10]. Contrary to this study, we assessed AVN occurrence before transplantation and distinguished it from AVN occurring after transplantation, limiting bias in the analysis of factors associated with AVN after transplantation, and thus, allowing better identification of AVN risk factors after KT. We also assessed the cumulative corticosteroid dose in the first year to improve the external validity of the study.

We confirmed that AVN developed early after transplantation in most patients (within two years). AVN most commonly affected the femoral head, but it was found across several sites in half of patients. Our study allowed us to determine the delay between the emergence of first symptoms and diagnosis. It also highlights a long diagnosis delay with a median of three months, limiting early management. The prognoses for half of these patients were particularly poor, requiring total hip replacement during the follow-up period. The difficulty of early diagnosis can be explained by the fact that AVN has subtle symptoms, with few disabling features during the early stages.

Interestingly–in order to improve the identification of high-risk patients, we found that a high body mass index (BMI) was a strong independent predictor for AVN after KT. Our finding is consistent with another KT-focused study [6] and a survey of the general population [24]. Previously, a study by Takao *et al*. failed to find an association between AVN and BMI in Japanese recipients, likely explained by the absence of overweight and obese patients in their cohort. High BMI may be involved in AVN development due to fatty infiltration of the bone marrow, resulting in elevated intraosseous extravascular pressure, diminished blood flow, and bone ischemia. Indeed, BMI is also a good measure for adiposity and correlates with bone marrow fat [25]. Furthermore, a study by Ferrari *et al*. suggested that obesity can lead to a decline in epiphysis blood flow as it associates with higher levels of fibrinolysis inhibitor [6]. Another possible underlying mechanism is the role of weight in generating subchondral fractures that lead to secondary ischemia. Weight-bearing sites are typically affected during AVN. Finally, a recent study using an animal model has indicated that leptin resistance during obesity is important to AVN pathogenesis [26]. This result strongly suggests that high BMI is a new predictive risk factor for AVN. Importantly, this risk factor is increasing in prevalence. Indeed, the mean BMI among patients receiving transplant in the United States has steadily increased from 25.5 kg/m^2^ to more than 28 kg/m^2^ between 1995 and 2009 [27].

In addition to BMI, the use of corticosteroids was still identified as a risk factor in the current study. Corticotherapy is traditionally considered as the main AVN risk factor, also showing a strong relationship with dose [13,14,28]. Consistent improvements in immunosuppressive regimens have led to progressively lower effective steroid doses, which were parallelled by a decrease in AVN incidence [7,13,29]. However, most recent studies have shown contradictory results–with some reports demonstrating that even the reduced doses of corticosteroids in use today continue to be associated with AVN occurrence [7,13,29]. In contast, other studies failed to find similar associations [17,30]. Unfortunately, recent data on cumulative corticosteroid doses are frequently unavailable [4,6,10]. Using a steroid-free maintenance regimen (methylprednisolone 500 mg administered intraoperatively followed by prednisone, 1 mg/kg on posttransplant day 1; tapering over 4 days and discontinued after day 5), Kwaja et al did not observe any case of avascular osteonecrosis in their sample of 349 kidney transplant recipients [31]. However, our current data indicate that the cumulative dose of corticosteroids remains a major risk factor for post-transplant AVN. In our study, the cumulative corticosteroid dose was more than 2-fold lower than that used in most recent publications [3,13,17]. Despite a low corticosteroid cumulative dose (most frequently required by the occurrence of biopsy-proven acute rejection), patients with acute rejection should be regarded as being at high risk of devoping AVN. It would be interesting to differentiate the prognostic effect of bolus dosing versus oral corticosteroid dose that is not clearly determined in the literature in all populations. In our study, we failed to discriminate the role of bolus vs. oral because these factors are highly correlated. Acute rejection is classically, and in our study, treated both by corticosteroid bolus and an increased oral dose of corticosteroid.

Moreover, to our knowledge, we are the first to specifically address the relation between hyperparathyroidism and osteonecrosis. An association was suggested [2,6] and found in a study which analyzed the PTH level in 215 patients transversally [6]. The PTH level decreased progressively after transplantation and varied according to the time of measurement after transplantation [19]. High persistent levels of PTH persist in about one third of patients one year after transplantation. To address this issue, we analyzed PTH levels prospectively determined at the time of transplantation and at three month post-transplantation and identified a significant association between secondary hyperparathyroidism and AVN. However, we did not find greater significance in this association in our multivariate analysis. This may be explained by an insufficient number of events or that secondary hyperparathyroidism was associated with being overweight [19]. Secondary hyperparathyroidism induces extensive bone marrow fibrosis [32] and may, therefore, lead to diminished blood flow. As hyperparathyroidism is a risk factor that contributes to fracture after KT [19], it could lead to small subchondral fractures in the third state of Arlet and Ficat classification [33], causing secondary ischemia. Similarly, we did not identify an increased risk in cases with pre-existing osteopenia or osteporosis at transplantation; this result is at variance with previous reports [8,12,15]. The lack of significant differences in our study can be attributed to the negligible impact of lower BMD on epiphyseal ischemia, which is responsible for AVN. We also did not find any association between dyslipidemia and AVN, contrary to some studies [16]. This may be due to statin treatment reducing AVN incidence [4,34].

Finally, our study did not find association between the use of CsA vs. tacrolimus and AVN after transplantation. This association was suggested in a recent cohort study [10]. Nevertheless, the authors do not mention on what criteria they chose tacrolimus vs. ciclosporin in their patients. The hypothesis that bone remodeling is increased more than tacrolimus is weak. This association may be explained by the fact that the rate of biopsy-proven acute rejection is lower in renal transplant patients receiving tacrolimus than in those receiving CsA [35] and that patients treated by CsA may have received more corticosteroids.

Our study has some limitations. Alcohol consumption, known to be related to AVN in the general [36] and KT populations [15], was not evaluated. Some previously recognized risk factors were not identified in our study, including hemostatic abnormalities, HIV seropositivity, and number of pregnancies, suggesting a possible lack of patients. Some asymptomatic AVN cases may have been missed. Previous screening studies using MRI indicated that AVN incidence among renal recipients ranges widely from 4 to 25%, depending on the corticosteroid dose administered [3,5,7,37]. However, a previous study by Ferrari *et al*. that screened 81 asymptomatic kidney recipients using MRI found no evidence of AVN [6]. This suggests that asymptomatic AVN cases are rare. [10].

Nevertheless, this study has enabled the identification of high-risk patients that have high BMI or have received treatment for acute rejection. These recipients would benefit from targeted radiological screening after transplantation or from determination of genetic factors that predispose them for steroid-induced osteonecrosis [7,38,39]. Indeed, early diagnosis is important to delay the progression of AVN and to prevent bone collapse. Medical management, biophysical treatments, and joint preserving procedures may be more effective in the earlier stages of disease [11]. Currently, the reduction or early withdrawal of corticosteroid therapy remains the only documented preventive treatment available for AVN [40]. However, corticosteroid sparing is not possible in situations of acute rejection or in high-risk immunological recipients. As such, other preventive treatments may be possible in these high-risk patients, although they are not well documented. They include reducing BMI before transplantation and avoiding alcohol. Some pharmacological treatments, such as statins [34] and bisphosphonates [11], are also of interest. Bisphosphonates have not yet been evaluated for AVN prevention but have been shown to diminish pain and to improve joint function of avascular necrosis of the femoral head [41,42]. However, evidence suggesting a reduction in joint collapse has not been demonstrated [43].

In conclusion, our study with a large and recent cohort of KT patients has revealed that high BMI and corticosteroid treatment for acute rejection are strong predictors of AVN after transplantation. Clinicians should, therefore, seek to screen this high-risk group for earlier diagnosis of AVN and to optimize treatment that prevents bone collapse and preserves joints.

## Supporting information

S1 Dataset Anonymized complete dataset. (XLS)

## Abbreviations

25OHD: 25-hydroxy-vitamin D or calcidiol
95CI: 95% confidence interval
ABMR: antibody-mediated rejection
AHR: adjusted hazard ratio
AVN: avascular osteonecrosis
BMD: bone mineral density
BMI: body mass index
CsA: cyclosporine A
eGFR: estimated glomerular filtration rate
HD: hemodialysis
HIV: human immunodeficiency virus
KT: kidney transplantation
M3, M12, M24: month 3, 12 or 24 post-transplantation
MDRD: modification of diet in renal disease
MMF: mycophenolate mofetil
MRI: magnetic resonance imaging
OR: odds ratio
PD: peritoneal dialysis
preT-AVN: pre-transplant AVN
NOAVN: new onset AVN
PTH: parathyroid hormone
SD: standard deviation
TCMR: T cell-mediated rejection
